# The Immunoregulatory Role of Myeloid-Derived Suppressor Cells in the Pathogenesis of Rheumatoid Arthritis

**DOI:** 10.3389/fimmu.2020.568362

**Published:** 2020-09-15

**Authors:** Lan Yan, Mingge Liang, Tong Yang, Jinyu Ji, Goutham Sanker Jose Kumar Sreena, Xiaoqiang Hou, Meiqun Cao, Zhitao Feng

**Affiliations:** ^1^Medical College of China Three Gorges University, Yichang, China; ^2^The Institute of Rheumatology, The First College of Clinical Medical Sciences, China Three Gorges University, Yichang, China; ^3^Shenzhen Institute of Geriatrics, Shenzhen Second People's Hospital, The First Affiliated Hospital of Shenzhen University, Shenzhen, China

**Keywords:** myeloid-derived suppressor cell, rheumatoid arthritis, immune cell, immune regulation, cell therapy

## Abstract

Myeloid-derived suppressor cells (MDSCs) are a group of cells that regulate the immune response and exert immunosuppressive effects on various immune cells. Current studies indicate that MDSCs have both anti-inflammatory effects and proinflammatory effects on rheumatoid arthritis (RA) and RA animal models. MDSCs inhibit CD4^+^ T cells, which secrete proinflammatory factors such as IFN-γ, IL-2, IL-6, IL-17, and TNF-α, by inhibiting iNOS, ROS, and IFN-γ and promoting the production of the anti-inflammatory factor IL-10. MDSCs can suppress dendritic cells by reducing MHC-II and CD86 expression, expand Treg cells *in vitro* through the action of IL-10, inhibit B cells through NO and PGE_2_, and promote Th17 cell responses by secreting IL-1β. As a type of osteoclast precursor cell, MDSCs can differentiate into osteoclasts through activation of the NF-κB pathway via IL-1α. Overall, our study reviews the research progress related to MDSCs in RA, focusing on the effects of MDSCs on various types of cells and aiming to provide ideas to help reveal the important role of MDSCs in RA.

Rheumatoid arthritis (RA) is a chronic inflammatory autoimmune disease mediated by a variety of immune cells that is mainly defined by the erosive destruction of the joints in the extremities. The basic pathological changes are the infiltration of inflammatory cells, the destruction of cartilage, and the erosion of bone (1). The pathogenesis involves the T cell activation pathway (2), B cells (3), macrophages, osteoclasts (OCs) (4), dendritic cells (DCs) (5), and so on. Since the body's immune system is a complex network-regulation system, it is composed of innate immunity and adaptive immunity and involves interactions and regulation among innate immune cells, antigen-presenting cells, adaptive immune cells, etc. Myeloid-derived suppressor cells (MDSCs), inhibitory cells expressing the markers CD11b and Gr-1, have been found to be abundant in infection (6), tumors (7), inflammation (8), and other diseases and negatively regulate the body's immune function. MDSCs are a special group of cells that regulate the immune response. They can exert their immunosuppressive effects on a variety of immune cells through different mechanisms, resulting in declines in the body's innate and adaptive immune functions and promoting the development and progression of diseases. There have been many reports showing that MDSCs have a strong immunosuppressive effect under abnormal conditions, but the roles of MDSCs and their subgroups in autoimmune arthritis are still controversial. Therefore, this article aims to review the role of MDSCs in the pathogenesis of RA and provide a theoretical basis for future research.

## Origin of MDSCs

MDSCs, which are composed of bone marrow progenitor cells and immature myeloid cells (IMCs), are a heterogeneous cell group composed of morphologically, phenotypically, and functionally diverse but also highly immunosuppressive myeloid cells (8). Under normal conditions, IMCs develop into mature DCs, macrophages, and granulocytes after being generated in the bone marrow and then participate in immune responses in specific target organs. However, under the pathological conditions of a tumor, inflammation, infection, trauma, autoimmune disease, etc., various factors can contribute to the formation of MDSCs; vascular endothelial growth factor (VEGF), granulocyte-macrophage colony-stimulating factor (GM-CSF), interleukin (IL)-4, and other cytokines can block IMC differentiation (9). IMCs inhibit their own differentiation by releasing immunosuppressive factors, such as arginase-1 (Arg-1), inducible nitric oxide synthase (iNOS), nitric oxide (NO), and reactive oxygen species (ROS), ensuring that myeloid precursor cells cannot mature. IMCs expand *in vivo*, migrate out of the bone marrow, and accumulate in the peripheral blood and spleen or lesions, where they form MDSCs (10–12). Initially, in the field of tumor biology, MDSCs were considered to be immunosuppressive cells related to tumor expansion that accumulated near tumors and in the peripheral blood, promoted immune escape by tumor cells, and accelerated disease progression (13, 14). Recent research has found that MDSCs play an important immunosuppressive role in various immune diseases, so research on MDSCs in autoimmune diseases has expanded (15, 16). Under pathological conditions, dilated MDSCs can be identified in the blood, surrounding lymphoid tissues, the spleen, cancerous tissues, and inflamed sites in the corresponding target organs. They can inhibit other immune cells through direct contact or cytokine secretion, which usually inhibits the immune response (17).

## Phenotypes of MDSCs

The phenotypes of MDSCs are very different in mice and humans. MDSCs lack the most basic surface recognition features due to blocked differentiation, so MDSCs are morphologically indistinguishable from granulocytes and monocytes (18).

In mice, MDSCs have specific surface markers and are defined as cells that coexpress the myeloid antigens CD11b and Gr-1. According to the morphology of this cell population and the difference in the expression levels of the two Gr-1 epitopes, Ly6G and Ly6C, they can be divided into two subsets: monocytic MDSCs (M-MDSCs) and granulocytic MDSCs (G-MDSCs), which have the phenotypes CD11b^+^Ly6G^−^Ly6C^high^ and CD11b^+^Ly6G^+^Ly6C^low^, respectively (19, 20). The subgroups of M-MDSCs and G-MDSCs can also be divided by CD49d and CD11b expression patterns, and their phenotypes are CD11b^+^Ly6G^+/−^Ly6C^high^CD49d^+^ and CD11b^+^Ly6G^+^Ly6C^low/−^CD49d^−^. Additionally, some surface molecules, such as IL-4Rα, F4/80, CD80, CD31, and CD115, can be used to recognize the inhibitory functions of MDSC subgroups (21).

In patients, MDSCs can also be divided into M-MDSCs and G-MDSCs. Because CD33 and CD11b are coexpressed in human subsets, which also express CD14 and CD15, respectively, the phenotypes of human M-MDSCs and G-MDSCs can be expressed as CD33^+^CD11b^+^CD14^+^ and CD33^+^CD11b^+^CD15^+^CD14^−^, respectively (22, 23). In addition, because MDSCs lack surface markers expressed by mature myeloid cells or lymphoid cells and express the MHC-II molecule HLA-DR, they can also be defined as CD33^+^CD11b^+^HLA-DR^−/low^. Due to their lack of lineage-specific antigens (Lin), such as CD3, CD19, and CD56, MDSCs are often described as Lin^−^CD11b^+^CD33^+^HLA-DR^−^ (24, 25). MDSCs express various surface markers and are divided into different subsets, probably because of different transcription factors and immunomodulatory molecules, such as cytokines, growth factors, and inflammatory mediators, which are presented in different disease microenvironments, blocking the normal differentiation of IMCs and thereby causing these cells to arrest in various stages of development. Therefore, MDSCs have different surface marker expression patterns at different stages. The complexity of these surface marker patterns leads to the heterogeneity of MDSCs. Thus, according to the actual pathological condition, MDSCs show different inhibitory capabilities and functional mechanisms (Tables 1, 2).

**Table 1:** Subpopulations and phenotypes of MDSCs in RA patients.

**Source**	**Sample**	**Subset**	**Phenotypes**	**Function**	**Reference**
RA patient	Synovial fluid	G-MDSCs	CD11b^+^CD33^+^HLA-DR^lo/−^CD14^−^CD15^+^	Inhibit T cells proliferation	(26)
	Synovial fluid	M-MDSCs	CD11b^+^CD33^+^HLA-DR^lo/−^CD14^+^CD15^−^	/	(26)
	Peripheral blood	G-MDSCs	CD14^−^HLA-DR^−^CD33^+^CD11b^+^	The proportion of MDSCs is negatively correlated with the proportion of Th1 cells	(27)
	Peripheral blood	G-MDSCs	CD14^−^HLA-DR^−^CD33^+^CD11b^+^	The proportion of MDSCs is negatively correlated with the proportion of Th1 cells	(28)
	Synovial fluid	/	CD11b^+^CD33^+^	Promote the differentiation of human Th17 cells *in vitro*	(29)
	Peripheral blood	MDSCs	CD11b^+^CD33^+^	Positive correlated with Th17 cells and RA activity	(30)
	Peripheral blood	MDSCs	CD14^−^HLA-DR^−^CD33^+^CD11b^+^	Th17 cells were negatively correlated with MDSCs	(31)
	Peripheral blood	/	CD11b^+^CD33^+^	MDSCs increased significantly in RA patients with high disease activity and promoted B cell proliferation *in vitro*	(32)

**Table 2:** Subpopulations and phenotypes of MDSCs in animal models.

**Source**	**Sample**	**Subset**	**Phenotypes**	**Function**	**Reference**
DBA/1J mice	Spleen	G-MDSCs	CD11b^+^Gr^−^1^high^	Reduction at an early stage, related to the expansion of Th17 cells	(30)
CIA model	Spleen	M-MDSCs	CD11b^+^Gr^−^1^medium^	Increased at a late stage, promotes Th17 cells differentiation *in vivo*	(30)
	Spleen	G-MDSCs	CD11b^+^Ly6C^+^Ly6G^+^	Inhibit T cells proliferation and Th1, Th17 cells differentiation	(25)
	Spleen	M-MDSCs	CD11b^+^Ly6C^+^Ly6G^−^	Moderately inhibits T cells proliferation, but its adoptive transfer does not affect Th1 and Th17 responses *in vivo*	(25)
	Bone marrow	M-MDSCs	CD11b^+^Ly6C^high^Ly6G^−^	Inhibit the proliferation of T cells, B cells	(33)
	Spleen	M-MDSCs	CD11c^−^CD11b^+^Ly6G^−^Ly6C^high^	IL-10-mediated reduction of joint inflammation after adoptive transfer	(34)
	Spleen	G-MDSCs	CD11c^−^CD11b^+^Ly6G^+^Ly6C^low^	Inhibit Th17 differentiation and promote Treg cells expansion	(34)
C57BL/6 mice	Spleen, Paw	M-MDSCs	CD11b^+^Ly6C^high^Ly6G^−^	Inhibit T cells proliferation and IFN-γ secretion, promote Th17 cells differentiation *in vitro*	(29)
CIA model		G-MDSCs	CD11b^+^Ly6C^low^Ly6G^+^	No effect of inhibiting T cells proliferation and IFN-γ secretion *in vitro*	(29)
BALB/c mice	Synovial fluid	G-MDSCs	Ly6G^high^Ly6C^int/low^	Inhibits DC maturation and specific T cells proliferation	(35)
PGIA model					

*RA, Rheumatoid Arthritis; CIA, Collagen-induced arthritis; PGIA, Proteoglycan-induced mouse arthritis model; G-MDSCs, granulocytic myeloid-derived suppressor cells; M-MDSCs, monocytic myeloid-derived suppressor cells; DC, dendritic cells*.

## Regulation Between MDSCs and Immune Cells

### MDSCs and CD4^+^ T Cells, Including Their Subpopulations

CD4^+^ T cells are a subset of lymphocytes that play an important role in specific immune responses. Antigen-presenting cells can activate self-reactive CD4^+^ T cells by presenting cognate antigens, which results in the T cells differentiating into various types of CD4^+^ helper T cells subpopulations, including Th1 cells, Th2 cells, and Th17 cells; these T cells can also differentiate into regulatory T cells (Treg cells) (36). T cells play an important role in the immune response involved in RA.

#### Regulatory Effect of MDSCs on CD4^+^ T Cells

Kurko et al. found that MDSCs exist in the synovial tissue of RA patients. Most of these MDSCs exhibit a neutrophil phenotype and morphology and can inhibit T cell infiltration in RA. This suggests that the increase in MDSC numbers observed in the synovial fluid (SF) of RA patients may be beneficial (26). Fujii et al. found that when the severity of arthritis in collagen-induced arthritis (CIA) mice peaked, MDSCs accumulated in the spleen; adoptive transfer of MDSCs into CIA mice could reduce the severity of disease and the numbers of CD4^+^ T cells and Th17 cells in the lymph nodes. MDSCs could also inhibit the proliferation of CD4^+^ T cells, their differentiation into Th17 cells *in vitro* and the production of proinflammatory factors secreted by CD4^+^ T cells such as IFN-γ, IL-2, IL-6, and TNF-α and promote the production of the anti-inflammatory factor IL-10 secreted by CD4^+^ T cells, which suggests that MDSCs play an important role in the regulation of CIA by inhibiting the proinflammatory response of CD4^+^ T cells (37). Crook et al. (33) found that in autoimmune arthritis, M-MDSCs inhibit the proliferation of autologous CD4^+^ T cells in the CIA model in a manner dependent on iNOS and IFN-γ. Egelston et al. (35) found that the synovial fluid of proteoglycan-induced arthritis (PGIA) mice contains a large number of MDSCs, which can inhibit T cell proliferation effectively through iNOS and ROS. Park et al. (34) found that MDSCs derived from CIA mice reduced IL-17 production and increased FOXP3 expression in CD4^+^ T cells *in vitro*. In RA-associated interstitial lung disease (RA-ILD), Sendo et al. (38) found that CD11b^+^Ly6C^high^ cells (M-MDSCs) isolated from the lungs could develop the CD11b^+^Gr-1^dim^ phenotype when cultured with GM-CSF and IL-4-producing cells and the CD11b^+^Gr-1^dim^ cells could inhibit T cell proliferation. In addition, lung MDSCs inhibit the proliferation of CD4^+^ T cells in an MDSC density-dependent manner and inhibit the differentiation of CD4^+^ T cells into Th17 cells (39).

Obviously, in RA patients, MDSCs can inhibit infiltrating T cells in the joints. In autoimmune arthritis mice, MDSCs mainly inhibit CD4^+^ T cells through iNOS, ROS, and IFN-γ. MDSCs can inhibit CD4^+^ T cell proliferation, differentiation into Th17 cells, and secretion of proinflammatory factors such as IFN-γ, IL-2, IL-6, IL-17, and TNF-α and promote the production of the anti-inflammatory factor IL-10. The production of IL-10 increases FOXP3 expression *in vitro*. These results suggest that MDSCs play a crucial role in the immune regulation occurring during RA.

#### Regulatory Effect of MDSCs on Th1 Cells

Th1 cells play an important role in the development of RA (40). Th1 cells and the signature cytokine IFN-γ play important roles in RA inflammation, and one of the methods to treat RA is to inhibit the Th1 response (41).

Studies have found that peripheral blood MDSCs in RA patients are positively correlated with disease activity and that the proportion of MDSCs is negatively correlated with the proportion of Th1 cells, which suggests that in the peripheral blood of RA patients, MDSCs and Th1 cells may be mutually antagonistic and participate in the development of RA together (27, 28). Park et al. (34) injected MDSCs into CIA mice *in vivo* and found that the number of Th1 cells in the spleen of the mice decreased, suggesting that MDSCs have an inhibitory effect on Th1 cells. Wang et al. (25) found that adoptive transfer of G-MDSCs into CIA mice reduces joint inflammation and the frequency of Th1 cells in the draining lymph node, suggesting that G-MDSCs have an inhibitory effect on Th1 cells in CIA that reduces joint inflammation.

All these studies have shown that MDSCs are negatively associated with Th1 cells in the peripheral blood of RA patients and the spleen of CIA mice. Further studies are required to fully understand the role of MDSCs in the regulation of Th1 cells during the pathological development of RA.

#### Regulatory Effect of MDSCs on Th17 Cells

Th17 cells are a subset of inflammatory CD4^+^ T cells that mainly secrete IL-17. Studies have found that Th17 cells are associated with many autoimmune diseases, including RA, psoriasis, and multiple sclerosis (42). Studies have also found that blocking the function of Th17 cells may inhibit the development of RA. As a class of immunosuppressive cells, MDSCs play an essential role in the development of RA, and recent studies have found that in RA, MDSCs have a regulatory effect on Th17 cells.

Guo et al. found that the frequency of MDSCs in the synovial tissue of RA patients was positively associated with the level of IL-17A. Additionally, the MDSCs of RA patients and CIA mice could both promote the differentiation of human Th17 cells *in vitro*, and MDSCs could promote Th17/IL-17 responses. The increase in Th17 cells infiltration that occurs during the progression of CIA affected the accumulation of MDSCs, and the removal of MDSCs reduced the frequency of Th17 cells in the spleen of CIA mice, which suggests that MDSCs are positively correlated with Th17 cells and that MDSCs have certain proinflammatory effects. In addition, M-MDSCs were more effective than G-MDSCs in promoting Th17 cell differentiation (29). Zhang et al. found that the numbers of CD14^+^HLA-DR^−/low^ cells in the peripheral blood of RA patients were significantly higher than those in healthy controls and that the expansion of CD14^+^HLA-DR^−/low^ cells was closely associated with Th17 cells and Disease Activity Score-28 (DAS28) results. In CIA mice, the depletion of MDSCs *in vivo* led to the inhibition of T cell proliferation and reductions in IL-17A and IL-1β production, while adoptive transfer of MDSCs could lead to increased disease severity in mice, including joint swelling, cell infiltration, bone erosion, and cartilage destruction, and significantly increased serum IL-17A and IL-1β levels, which suggests that MDSCs play important roles in the development of RA and CIA (30). Studies have found that MDSCs are the main source of IL-1β, that MDSCs in CIA mice can express high levels of IL-1β and that MDSCs promote the differentiation of Th17 cells and CD4^+^ T cells via the IL-1β signaling pathway, which suggests that MDSCs in CIA mice can promote the Th17 cell response through high expression of IL-1β (29, 30, 43). Cheng et al. found that the proportion of CD11b^+^Gr1^+^ MDSCs in their CIA group was positively correlated with the proportion of Th17 cells. After coculturing MDSCs and CD4^+^ T cells, the proinflammatory factor IL-1β was highly expressed, while after blocking IL-1β, Th17 cell numbers, and IL-17A, STAT3, and RORγt mRNA expression levels were significantly reduced, which suggests that MDSCs, as a potential source of IL-1β, have a proinflammatory effect, mediating CD4^+^ T cell differentiation into Th17 cells (44). Jiao et al. (31) found that in the peripheral blood of RA patients, Th17 cells were negatively correlated with MDSCs. Li et al. (45) noted that G-MDSCs could suppress the production of Th17 cells by secreting exosomes to ameliorate the pathology of CIA mice. Studies have found that adoptive transfer of MDSCs is beneficial in autoimmune arthritis and can reduce the number of Th17 cells in the draining lymph nodes and joint tissues, thereby reducing joint inflammation (25, 37, 46).

The above research shows that MDSC numbers are increased in the peripheral blood of RA patients, which promotes increases in the levels Th17 cells, IL-17A, and IL-1β. MDSCs can enhance the response of Th17 cells by secreting IL-1β, which suggests that MDSCs are mainly responsible for promoting the inflammatory effect. However, another study found that MDSCs were negatively correlated with Th17 cells in the peripheral blood of RA patients, suggesting that MDSCs can reduce the number of Th17 cells to relieve joint inflammation. MDSCs can promote Th17 cell responses in RA, mainly by secreting IL-1β, but MDSCs can also have an inhibitory effect on Th17 cells; the specific mechanism is not clear. Therefore, further research on the regulatory effect of MDSCs on Th17 cells in RA could clarify the role of MDSCs in RA.

#### Regulatory Effect of MDSCs on Treg Cells

In RA, Treg cells can be recruited to inflamed joints to exert a local inhibitory effect, which leads to increased levels of Treg cells in the joint synovial fluid and decreased levels in the peripheral blood (47). Some studies have found that MDSCs can participate in the development of RA by regulating Treg cells.

Park et al. showed that the number of Tregs in the spleen of mice treated with MDSCs increased and that the injection of G-MDSCs into CIA mice could promote Treg cell proliferation and weaken the joint inflammation in the mice. Treg cells were expanded *in vitro* in the presence of an anti-IL-10 antibody, and this antibody blocked the expansion effect of MDSCs on Treg cells, suggesting that without IL-10, MDSCs cannot inhibit joint inflammation. This may suggest that IL-10 plays an important role in the enhancement of Treg responses by MDSCs (34).

The above research suggests that G-MDSCs can promote the proliferation of Treg cells in CIA mice and that via IL-10, MDSCs can promote the proliferation of Treg cells *in vitro*. Further studies on the interaction among MDSCs, IL-10, and Treg cells in RA may help better clarify the role of MDSCs in RA.

### Regulatory Effect of MDSCs on B Cells

B cells play a major role in the pathogenesis of RA, which not only promotes the production of autoantibodies but also regulates the function of T cells and DCs and promotes the development of ectopic lymphoid neoplasia and release of inflammatory mediators (48). At present, B cell-targeted therapy is effective in early and late established RA (49). Some studies have found that MDSCs have a regulatory effect on B cells in RA.

Crook et al. (33) showed that M-MDSCs in CIA mouse bone marrow could inhibit the proliferation and activation of B cells and produce specific antibodies through nitric oxide (NO) and prostaglandin E2 (PGE_2_) to suppress the immune response. Another analysis of the peripheral blood of RA patients found that MDSC numbers were significantly increased in RA patients with high disease activity and these MDSCs promoted B cells proliferation *in vitro* (32).

MDSCs can inhibit B cells via NO and PGE_2_ in the CIA model and promote B cell expansion in the peripheral blood of RA patients. The difference in effect may be due to the different sources of MDSCs, but further research is still needed. Overall, MDSCs may participate in the development of RA through B cell regulation, but the specific regulation of B cells by MDSCs still needs to be further explored.

### Regulatory Effect of MDSCs on Macrophages

One of the typical symptoms of RA is inflammation caused by the accumulation of fibroblasts, lymphocytes, neutrophils, and monocytes/macrophages. Among these cells, activated macrophages are the main source of proinflammatory cytokines and chemokines, including TNF-α, IL-6, CXCL4, and CXCL7. Activated macrophages can activate endothelial cells and induce inflammation in the synovium and the production of OCs, eventually leading to joint damage (50, 51). In the pathogenesis of RA, macrophages play an important role. The increase in the number of macrophages in the synovium is considered an early biomarker of RA, and numerical changes can be used to distinguish effective treatment, ineffective treatment, and placebo treatment (52). Some studies have found that in the process of RA, MDSCs can regulate macrophages.

To evaluate the effect of MDSCs on macrophages *in vivo*, Zhang et al. (46) examined the frequency of macrophages in the draining lymph nodes and joint tissues of CIA mice treated with a phosphate-buffered saline (PBS) solution and MDSCs and found that the numbers of CD11b^+^CD68^+^ macrophages in the CIA mice treated with MDSCs were significantly reduced in the draining lymph nodes and joint tissues, suggesting that adoptive transfer of MDSCs can reduce the degrees of arthritis and histological damage in the CIA model by suppressing macrophages.

Macrophages play a key role in the pathological development of RA, and the regulatory mechanism by which MDSCs affect macrophages is currently uncertain. Therefore, further research on the MDSC-mediated regulatory mechanism modulating macrophages in RA may provide new ideas to clarify the specific roles of MDSCs in RA and the treatment of RA.

### Regulatory Effect of MDSCs on DCs

DCs are important innate immune cells and professional antigen-presenting cells. They play a vital role in the initiation of immunity. Some studies have found that DCs play an important role in the pathological process of RA (53). Studies have found that MDSCs have a regulatory effect on DCs.

To study the possible effects of synovial fluid (SF) cells on DC maturation, Egelston et al. observed MHC-II and CD86 expression by DCs cultured alone or in the presence of SF cells (90% Gr-1^+^CD11b^+^ myeloid cells with a neutrophil morphology). The levels of MHC-II and CD86 in the DCs were significantly reduced by coculture with SF cells compared to culturing without SF cells after 24 h. This may suggest that Gr-1^+^CD11b^+^ SF cells significantly reduce the expression levels of MHC-II and CD86, both of which play key roles in antigen presentation by DCs, while the results for Gr-1^+^CD11b^+^ cells also indicate that the SF cells of PGIA mice have characteristics of MDSCs, suggesting that MDSCs inhibit the maturation and activation of DCs *in vitro* (35).

The SF cells of arthritic joints in PGIA mice appear to have characteristics of MDSCs and can inhibit DCs by reducing the expression of MHC-II and CD86. Further investigations on the immunomodulatory effects of MDSCs on DCs may provide new ideas related to the immunomodulatory functions of MDSCs in RA.

### Regulatory Effect of MDSCs on OCs

Bone erosion is a sign of severe RA. Studies have found that OCs play a major role in bone resorption. Increased OC numbers or activity often leads to cartilage and bone destruction (54), and cytokines such as IL-1α, IL-1β, IL-6, IL-11, TNF-α, and M-CSF can provide signals for OC differentiation and bone resorption (55). Some studies have found that MDSCs are one of the types of OC precursor cells.

Sawant et al. cocultured MDSCs isolated from the bone marrow of breast cancer bone metastatic tumor-bearing mice with M-CSF and RANKL and stained the cells with TRAP to observe the expression of F4-80 during OC differentiation. They found that MDSCs are a new group of true OC progenitor cells and that MDSCs differentiate into OCs in a manner dependent on NO and have bone destruction function both *in vivo* and *in vitro*, suggesting that targeting MDSCs in breast cancer patients may reduce primary tumor growth and bone metastasis growth (56). Su et al. (57) found that in periodontitis, *Porphyromonas gingivalis* can induce the expansion of three subpopulations of MDSCs (Ly6G^++^Ly6C^+^, Ly6G^+^Ly6C^++^, and Ly6G^+^Ly6C^+^), and the CD11b^+^Ly6G^+^Ly6C^++^ subpopulation can differentiate into OCs and exert inhibitory effects on T cells, which suggests that MDSCs not only have an immunosuppressive effect but also promote OC development.

Recent studies have found that MDSCs, as precursor cells of OCs, play an important role in the development of autoimmune arthritis. Zhang et al. found that compared with that in the bone marrow of normal mice, the number of MDSCs in the bone marrow of CIA mice was significantly increased and that the CIA MDSCs were more likely to differentiate into OCs and contribute to bone resorption, thereby causing bone destruction. When MDSCs were cocultured with M-CSF and RANKL, they differentiated into OCs, while differentiation was inhibited when they were cocultured with an inhibitor of NF-κB. These results suggest that NF-κB plays an important role in the differentiation of MDSCs into OCs and that IL-1α activates the NF-κB pathway. Furthermore, when OC differentiation medium is supplemented with IL-1α (10 ng/ml), the differentiation of MDSCs into OCs is enhanced, suggesting that IL-1α can activate the NF-κB pathway to promote OC differentiation (58).

It is clear that MDSCs can differentiate into OCs in tumors and inflammatory diseases and have bone destruction functions *in vivo*. In CIA mice, MDSCs are one of the types of OC precursor cells. In the context of culture with M-CSF and RANKL, IL-1α can activate the NF-κB pathway to promote MDSC differentiation into OCs, resulting in an increased degree of bone destruction. Whether bone destruction is related to the number of circulating MDSCs and whether MDSCs can be used as a biomarker to evaluate the aggressiveness of RA still need further study.

## Conclusion

Similar reviews have also studied the roles of MDSCs in autoimmune arthritis. Li et al. (45) reviewed the effects and actions of MDSC subpopulations during the development of autoimmune arthritis and reported that both MDSC subpopulations play important roles in regulating the proliferation, response, and differentiation of CD4^+^ T cells during the progression of autoimmune arthritis. Rajabinejad et al. (59) described the functions of MDSCs and the relationship between MDSCs and inflammation in RA, concluding that there are two different hypotheses related to MDSC function in RA: MDSCs can exert a proinflammatory effect by increasing the number of Th17 cells, but MDSCs can also increase the population of Tregs to produce an anti-inflammatory effect. In our review, we have integrated the literature on the regulatory effects of MDSCs on immune cells in the field of RA, describing the functions of MDSCs in arthritis model mice and RA patients and focusing on the effects of MDSCs on various types of cells. We report that the proinflammatory and anti-inflammatory functions of MDSCs are not only mediated by increasing the number of Th17 cells and the number of Tregs, respectively.

**Figure 1:**
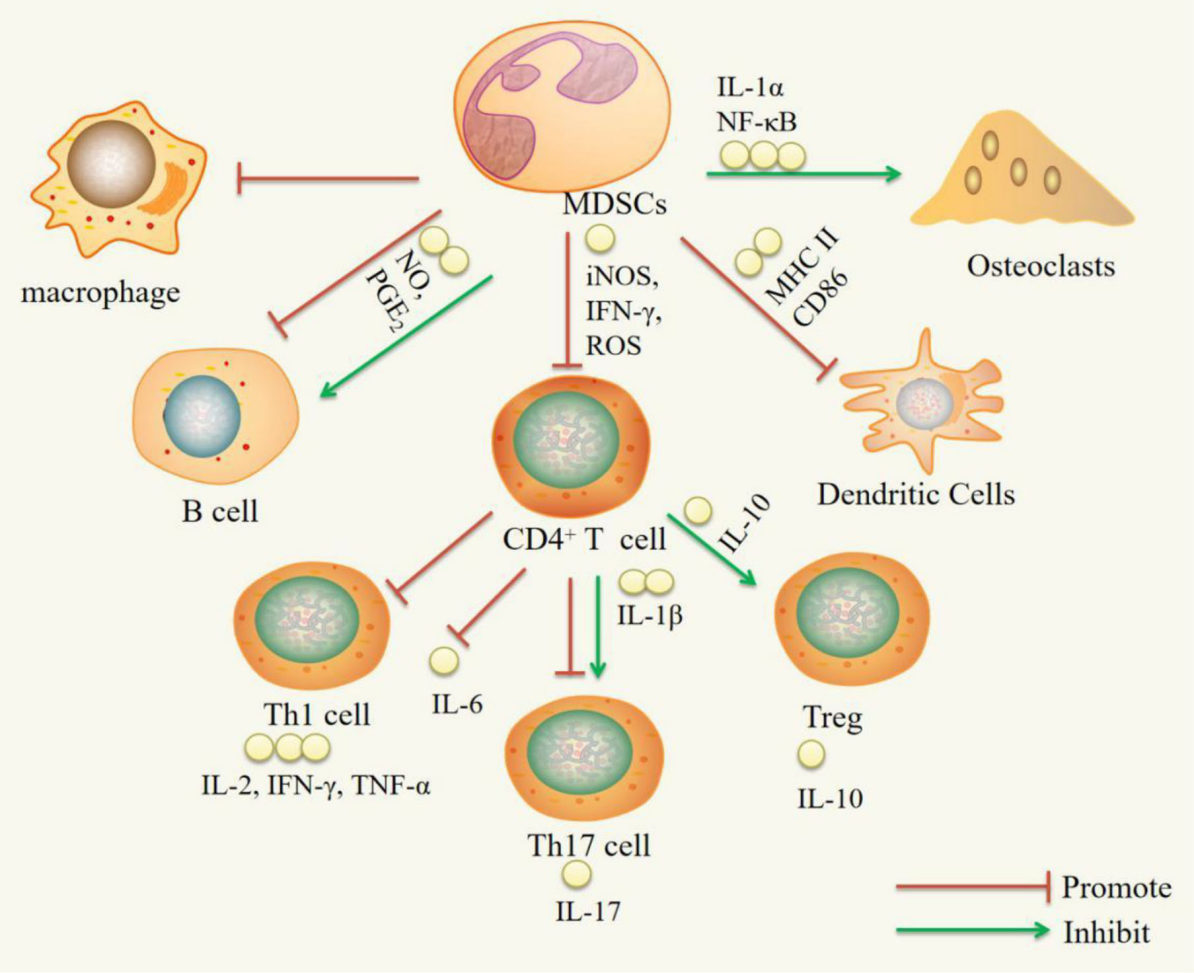
Immuregulatory effect of MDSCs on immune cells. IL-1α, interleukin-1α; NF-κB, nuclear transcription factor kappa B; iNOS, inducible nitric oxide synthase; IFN-γ, interferon-γ; ROS, reactive oxygen species; NO, nitric oxide; PGE2, Prostaglandin E2; IL-1β, Interleukin-1β; IL-10, Interleukin-10; IL-2, Interleukin-2; IL-6, Interleukin-6; IL-17, Interleukin-17; TNF-α, Tumor Necrosis Factor-α.

MDSCs have both proinflammatory and anti-inflammatory functions in RA and RA animal models. The proinflammatory function is mainly supported by the following observations: MDSCs can increase Th17 cell, B cell, and OC activity. MDSCs can promote Th17 cell responses by secreting IL-1β. As precursors of OCs, MDSCs can differentiate into OCs via NF-κB pathway signaling activated by IL-1α. The anti-inflammatory effect is mainly supported by the following observations: MDSCs can inhibit CD4^+^ T cells, Th1 cells, Th17 cells, B cells, macrophages, and DCs and promote Treg cell expansion. MDSCs inhibit CD4^+^ T cells, which secrete proinflammatory factors such as IFN-γ, IL-2, IL-6, IL-17, and TNF-α, by inhibiting iNOS, ROS, and IFN-γ and promote the production of the anti-inflammatory factor IL-10. MDSCs can suppress DCs by reducing MHC-II and CD86 expression. MDSCs can expand Treg cells *in vitro* through the action of IL-10. MDSCs can inhibit B cells through NO and PGE_2_ (Figure 1).

However, there are no reports on the relationship between MDSCs and NK cells in RA, and Nausch et al. (60) found that in RMA-S tumor mice, M-MDSCs could express retinoic acid early inducible-1 (RAE-1) ligand to interact with NKG2D ligands on NK cells and activate NK cells to produce large amounts of IFN-γ. This suggests that MDSCs have a regulatory effect on NK cells and that studying the interaction between MDSCs and NK cells may provide new ideas related to the mechanism involving MDSCs in the pathological process of RA.

MDSCs are IMCs and have an inhibitory effect on the antitumor immune response. MDSCs play a key role in maintaining immunosuppression under chronic inflammatory conditions, so inhibition of MDSC expansion and activation by MDSC-targeted agents may increase the efficiency of the immune system. At present, many drugs targeting MDSCs have been applied for tumor treatment, and the immunosuppressive effects of these drugs have also been shown to affect autoimmune diseases (61). Nishimura et al. (62) found that the JAK inhibitor tofacitinib could significantly promote the proliferation of MDSCs in the bone marrow of SKG mice and improve the arthritic process. In other autoimmune diseases, MDSCs have been found to have a regulatory effect on immune cells. Iwata et al. (63) found that in the MRL-Faslpr lupus mouse model, CD11b^+^Gr-1^low^ cells inhibited the proliferation of CD4^+^ T cells through Arg-1, and the percentage of CD11b^+^Gr-1^low^ cells was increased in the spleen, kidneys, and blood of 10-week-old lupus mice, suggesting that these cells contribute to immune regulation. Knier et al. (64) found that in experimental autoimmune encephalomyelitis, Ly6G^+^ neutrophils differentiated into MDSCs in the central nervous system of wild-type mice in a STAT3-dependent manner, controlling the accumulation and activation of B cells in this compartment, and therapeutic interventions that modulate the interaction of MDSCs with B cells might prevent the continuation of the inflammatory response in the central nervous system compartment in chronic autoimmune diseases (where local aggregates of B cells are drivers of immunopathology) (64).

In conclusion, existing studies have suggested that MDSCs play an important role in RA. The heterogeneity, plasticity, and multiple phenotypes of MDSCs regulate T cells, B cells, DCs, OCs, macrophages, and other cells through various mechanisms to influence the immune response. However, given the existing mechanisms, it is difficult to provide a comprehensive hypothesis to explain the specific role of MDSCs. The differentiation, expansion, and migration of MDSCs are also constrained by many factors, so cell therapy also faces many obstacles (17). Understanding the inherent multifunctional nature of MDSCs and the ability to influence organ-specific targets will help elucidate the mechanisms of autoimmune diseases and possible new treatments (65). Therefore, further study of the mechanism of action of MDSCs in RA may provide new ideas for the diagnosis, treatment, and prognosis of RA.

## Author Contributions

ZF and MC conceived the review. LY, ML, TY, JJ, and XH searched databases and drafted the manuscript. ZF, GJ, and MC amended the final manuscript. All authors contributed to the article and approved the submitted version.

## Conflict of Interest

The authors declare that the research was conducted in the absence of any commercial or financial relationships that could be construed as a potential conflict of interest.
